# The diagnostic accuracy of serological tests for Lyme borreliosis in Europe: a systematic review and meta-analysis

**DOI:** 10.1186/s12879-016-1468-4

**Published:** 2016-03-25

**Authors:** M. M. G. Leeflang, C. W. Ang, J. Berkhout, H. A. Bijlmer, W. Van Bortel, A. H. Brandenburg, N. D. Van Burgel, A. P. Van Dam, R. B. Dessau, V. Fingerle, J. W. R. Hovius, B. Jaulhac, B. Meijer, W. Van Pelt, J. F. P. Schellekens, R. Spijker, F. F. Stelma, G. Stanek, F. Verduyn-Lunel, H. Zeller, H. Sprong

**Affiliations:** VU University Medical Center, PO Box 7057, 1007 MB Amsterdam, The Netherlands; Canisius-Wilhelmina Hospital, PO Box 9015, 6500 GS Nijmegen, The Netherlands; National Institute for Public Health and the Environment (RIVM), Antonie van Leeuwenhoeklaan 9, 3721 MA Bilthoven, The Netherlands; European Centre for Disease Prevention and Control (ECDC), 171 83 Stockholm, Sweden; Izore Centre for Infectious Diseases Friesland, PO Box 21020, 8900 JA Leeuwarden, The Netherlands; HagaZiekenhuis, Leyweg 275, 2545 CH The Hague, Netherlands; Department of Medical Microbiology, Onze Lieve Vrouwe Gasthuis, P.O. Box 95500, 1090 HM Amsterdam, The Netherlands; Slagelse Hospital, Fælledvej 1, 4200 Slagelse, Region Zealand Denmark; German National Reference Centre for Borrelia, Bavarian Health and Food Safety Authority, Veterinärstraße 2, 85764 Oberschleißheim, Germany; Centre for Experimental and Molecular Medicine, Academic Medical Center, Amsterdam, The Netherlands; National Reference Centre for Borrelia, Department Laboratory of Bacteriology, Strasbourg University Hospital, 1 Place de l’Hôpital, Strasbourg, France; Department of Clinical Epidemiology, Biostatistics and Bioinformatics, Academic Medical Center, University of Amsterdam, PO Box 22700, 1100 DE Amsterdam, The Netherlands; Laboratory for Infectious Diseases, PO Box 30039, 9700 RM Groningen, The Netherlands; Dutch Cochrane Centre, Julius Center for Health Sciences and Primary Care/University Medical Center, PO Box 85500, 3508 GA Utrecht, The Netherlands; Radboud University Nijmegen Medical Centre, Geert Grooteplein-Zuid 10, 6525 GA Nijmegen, The Netherlands; Institute for Hygiene and Applied Immunology, Medical University of Vienna, Vienna, Austria; Department of Medical Microbiology University Medical Center Utrecht (UMC), P.O. Box 85500, 3508GA Utrecht, The Netherlands

**Keywords:** Lyme borreliosis, serology, sensitivity and specificity, meta-analysis

## Abstract

**Background:**

Interpretation of serological assays in Lyme borreliosis requires an understanding of the clinical indications and the limitations of the currently available tests. We therefore systematically reviewed the accuracy of serological tests for the diagnosis of Lyme borreliosis in Europe.

**Methods:**

We searched EMBASE en MEDLINE and contacted experts. Studies evaluating the diagnostic accuracy of serological assays for Lyme borreliosis in Europe were eligible. Study selection and data-extraction were done by two authors independently. We assessed study quality using the QUADAS-2 checklist. We used a hierarchical summary ROC meta-regression method for the meta-analyses. Potential sources of heterogeneity were test-type, commercial or in-house, Ig-type, antigen type and study quality. These were added as covariates to the model, to assess their effect on test accuracy.

**Results:**

Seventy-eight studies evaluating an Enzyme-Linked ImmunoSorbent assay (ELISA) or an immunoblot assay against a reference standard of clinical criteria were included. None of the studies had low risk of bias for all QUADAS-2 domains. Sensitivity was highly heterogeneous, with summary estimates: erythema migrans 50 % (95 % CI 40 % to 61 %); neuroborreliosis 77 % (95 % CI 67 % to 85 %); acrodermatitis chronica atrophicans 97 % (95 % CI 94 % to 99 %); unspecified Lyme borreliosis 73 % (95 % CI 53 % to 87 %). Specificity was around 95 % in studies with healthy controls, but around 80 % in cross-sectional studies. Two-tiered algorithms or antibody indices did not outperform single test approaches.

**Conclusions:**

The observed heterogeneity and risk of bias complicate the extrapolation of our results to clinical practice. The usefulness of the serological tests for Lyme disease depends on the pre-test probability and subsequent predictive values in the setting where the tests are being used. Future diagnostic accuracy studies should be prospectively planned cross-sectional studies, done in settings where the test will be used in practice.

## Background

Lyme borreliosis is one of the most prevalent vector-borne diseases in Europe. Its incidence varies between countries, with approximately 65,500 patients annually in Europe (estimated in 2009) [1]. It is caused by spirochetes of the *Borrelia burgdorferi* sensu lato species complex, which are transmitted by several species of Ixodid ticks [2]. In Europe, at least five genospecies of the *Borrelia burgdorferi* sensu lato complex can cause disease, leading to a variety of clinical manifestations including erythema migrans (EM), neuroborreliosis, arthritis and acrodermatitis chronica atrophicans (ACA). Each of these clinical presentations can be seen as a distinct target condition, i.e. the disorder that a test tries to determine, as they affect different body parts and different organ systems, and because the patients suffering from these conditions may enter and travel through the health care system in different ways, hence following different clinical pathways.

The diagnosis of Lyme borreliosis is based on the presence of specific symptoms, combined with laboratory evidence for infection. Laboratory confirmation is essential in case of non-specific disease manifestations. Serology is the cornerstone of Lyme laboratory diagnosis, both in primary care and in more specialized settings. Serological tests that are most often used are enzyme-linked immunosorbent assays (ELISAs) or immunoblots. ELISAs are the first test to be used; immunoblots are typically applied only when ELISA was positive. If signs and symptoms are inconclusive, the decision may be driven by the serology test results. In such a situation, patients may be treated with antibiotics after a positive serology result – a positive ELISA possibly followed by a positive immunoblot. After negative serology – a negative ELISA or a positive ELISA followed by a negative immunoblot – patients will not be treated for Lyme borreliosis, but they will be followed up or referred for further diagnosis. This implies that false positively tested patients (who have no Lyme borreliosis, but have positive serology) will be treated for Lyme borreliosis while they have another condition. It also implies that falsely negative tested patients (who have the disease, but test negative) will not be treated for Lyme borreliosis. A test with a high specificity – which is the percentage true negative results among patients without the target condition – will result in a low percentage of false positives. A test with a high sensitivity – being the percentage true positives among patients with the target condition – will result in a low percentage of false negatives.

The interpretation of serology results is complicated. The link between antibody status and actual infection may not be obvious: non-infected people may have immunity and test positive, while infected people may have a delay in their antibody response and may test negative. Furthermore, there is an overwhelming number of different available assays that have all been evaluated in different patient populations and settings and that may perform differently for the various disease manifestations [3]. We therefore systematically reviewed all available literature to assess the accuracy (expressed as sensitivity and specificity) of serological tests for the diagnosis of the different manifestations of Lyme borreliosis in Europe. Our secondary aim was to investigate potential sources of heterogeneity, for example test-type, whether the test was a commercial test or an in-house test, publication year and antigens used.

## Methods

We searched EMBASE and Medline (Appendix 1) and contacted experts for studies evaluating serological tests against a reference standard. The reference standard is the test or testing algorithm used to define whether someone has Lyme borreliosis or not. We included studies using any reference standard, but most studies used clinical criteria, sometimes in combination with serology. Studies performed in Europe and published in English, French, German, Norwegian, Spanish and Dutch were included.

The ideal study type to answer our question would be a cross-sectional study, including a series of representative, equally suspected patients who undergo both the index test and the reference standard [4]. Such studies would provide valid estimates of sensitivity and specificity and would also directly provide estimates of prevalence and predictive values. However, as we anticipated that these cross-sectional studies would be very sparse, we decided to include case-control studies or so-called two-gate designs as well [5]. These studies estimate the sensitivity of a test in a group of cases, i.e. patients for whom one is relatively sure that they have Lyme borreliosis. They estimate the specificity in a group of controls, i.e. patients of whom one is relatively sure that they do not have Lyme borreliosis. These are healthy volunteers, or patients with other diseases than Lyme.

We included studies on ELISAs, immunoblots, two-tiered testing algorithms of an ELISA followed by an immunoblot, and specific antibody index measurement (calculated using the antibody titers in both serum and cerebrospinal fluid). We excluded indirect fluorescent antibody assays, as these are rarely used in practice. Studies based on make-up samples were excluded. We also excluded studies for which 2 × 2 tables could not be inferred from the study results.

For each article, two authors independently collected study data and assessed quality. We assessed the quality using the Quality Assessment of Diagnostic Accuracy Studies-2 (QUADAS-2) checklist. This checklist consists of four domains: patient selection, index test, reference standard and flow and timing [6]. Each of these domains has a sub-domain for risk of bias and the first three have a sub-domain for concerns regarding the applicability of study results. The sub-domains about risk of bias include a number of signalling questions to guide the overall judgement about whether a study is highly likely to be biased or not (Appendix 2).

We analysed test accuracy for each of the manifestations of Lyme borreliosis separately and separately for case-control designs and cross-sectional designs. If a study did not distinguish between the different manifestations, we used the data of this study in the analysis for the target condition “unspecified Lyme”. Serology assays measure the level of immunoglubulins (Ig) in the patient’s serum. IgM is the antibody most present in the early stages of disease, while IgG increases later in the disease. Some tests only measure IgM, some only IgG and some tests measure any type of Ig. In some studies, the accuracy was reported for IgM only, IgG only and for detection of IgG and IgM. In those cases, we included the data for simultaneous detection of both IgG and IgM (IgT).

We meta-analyzed the data using the Hierarchical Summary ROC (HSROC) model, a hierarchical meta-regression method incorporating both sensitivity and specificity while taking into account the correlation between the two [7]. The model assumes an underlying summary ROC curve through the study results and estimates the parameters for this curve: accuracy, threshold at which the tests are assumed to operate and shape of the curve. Accuracy is a combination of sensitivity and specificity; the shape of the curve provides information about how accuracy varies when the threshold varies. From these parameters we derived the reported sensitivity and specificity estimates. We used SAS 9.3 for the analyses and Review Manager 5.3 for the ROC plots.

There is no recommended measure to estimate the amount of heterogeneity in diagnostic accuracy reviews, but researchers are encouraged to investigate potential sources of heterogeneity [7]. The most prominent source of heterogeneity is variation in threshold, which is taken into account by using the HSROC model. Other potential sources of heterogeneity are: test type (ELISA or immunoblot); a test being commercial or not; immunoglobulin type; antigen used; publication year; late versus early disease; and study quality. These were added as covariates to the model to explain variation in accuracy, threshold or shape of the curve.

Some studies reported results for patients with “possible Lyme” (i.e. no clear cases, neither clear controls). We included these as cases. As this may lead to underestimation of sensitivity, we investigated the effect of this approach. Borderline test results were included in the test-positive group.

## Results

### Selection and quality assessment

**Fig. 1:**
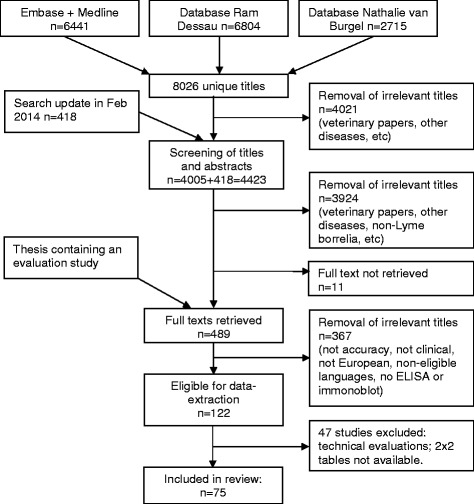
Results of the search and selection process

Our initial search in January 2013 retrieved 8026 unique titles and a search update in February 2014 revealed another 418 titles. After careful selection by two authors independently (ML, HS) we read the full text of 489 studies, performed data-extraction on 122 studies and finally included 75 unique published articles (Fig. 1). Fifty-seven of these had a case-control design, comparing a group of well-defined cases with a group of healthy controls or controls with diseases that could lead to cross-reactivity of the tests [8–64]. Eighteen had a cross-sectional design in which a more homogeneous sample of patients underwent both the serological assay(s) and the reference standard [65–82]. Three studies were not used in the meta-analyses, either because they used immunoblot as a reference standard [76, 79], or included asymptomatic cross-country runners with high IgG titers as controls [47].

**Fig. 2:**
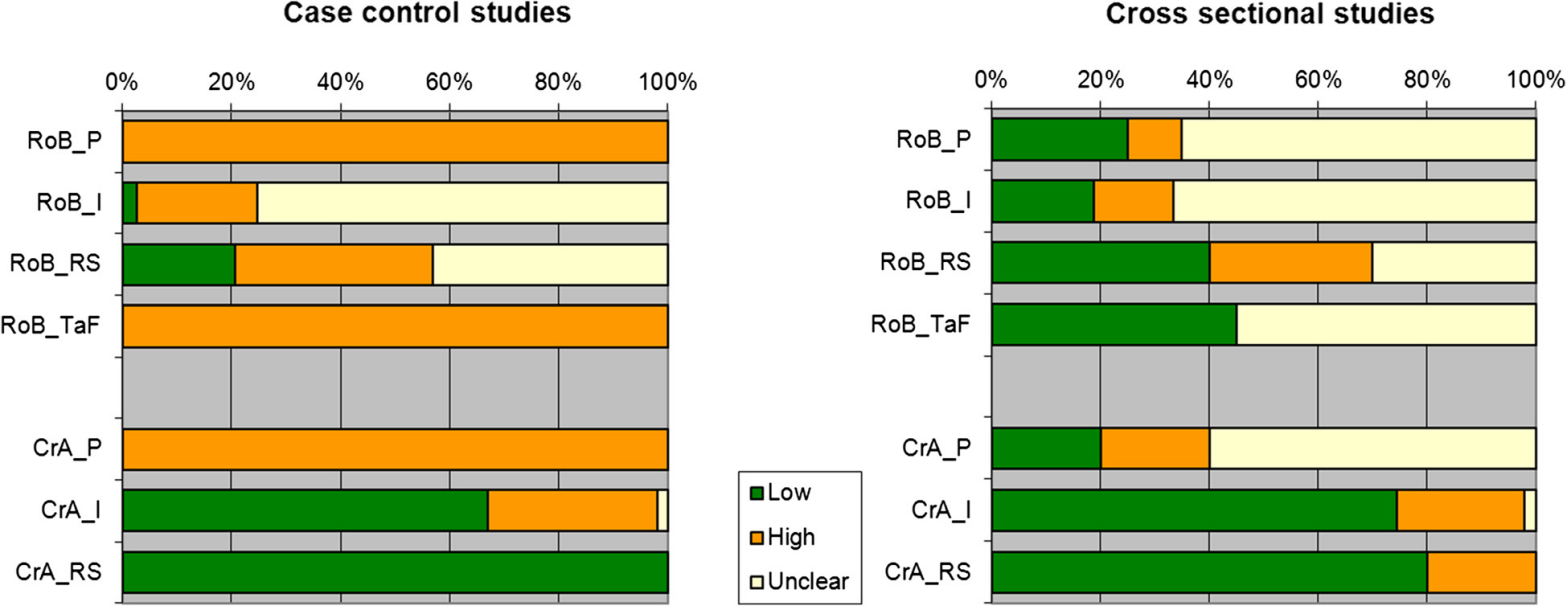
Methodological quality graph. Review authors’ judgements about each methodological quality item presented as percentages across all included studies. On the left-hand side the judgements for the included case control studies; and on the right-hand side those for the included cross-sectional studies. RoB: Risk of Bias; CrA: Concerns regarding applicability; P: patient sampling; I: Index test; RS: Reference Standard; TaF: timing and flow

None of the studies had low risk of bias in all four QUADAS-2 domains (Fig. 2 and Tables 1 and 2). Forty-six out of the 57 case-control studies and six out of the 18 cross-sectional studies scored unclear or high risk of bias in all four domains. All case-control studies had a high risk of bias for the patient sampling domain, because these designs exclude all “difficult to diagnose” patients [83]. Only three studies reported that the assessment of the index test was blinded for the disease status of the participants [45, 66, 75]. The cut-off value to decide whether a test is positive or negative was often decided after the study was done, which may also lead to bias in the index test domain [84]. The most common problem was inclusion of the serology results in the reference standard. The flow and timing domain was problematic in all case-control studies, as the cases and controls are usually verified in different ways. Three studies reported potential conflict of interest [31, 39, 62]. Most studies had a high concern regarding applicability, which means that either the included patients or the test used are not representative for clinical practice. Only three studies were representative for all domains [65, 73, 81].

**Table 1:** Quality assessment of included case control studies

Author Year	Design	RoB_P	CrA_P	RoB_I	CrA_I	RoB_RS	CrA_RS	RoB_TaF
Ang 2011	Case control	High	High	Unclear	Low	Unclear	Low	High
Ang 2012	Case control	High	High	Unclear	Low	Unclear	Low	High
Bergstrom 1991	Case control	High	High	High	High	High	Low	High
Branda 2013	Case control	High	High	Unclear	Low	High	Low	High
Cerar 2006	Case control	High	High	Unclear	Low	Low	Low	High
Cerar 2010	Case control	High	High	Unclear	Low	Unclear	Low	High
Christova 2003	Case control	High	High	Unclear	Low	Unclear	Low	High
Cinco 2006	Case control	High	High	Unclear	Low	Unclear	Low	High
Dessau 2010	Case control	High	High	Unclear	Low	High	Low	High
Dessau 2013	Case control	High	High	Unclear	Low	High	Low	High
Flisiak 1996	Case control	High	High	Unclear	Low	Low	Low	High
Flisiak 1998	Case control	High	High	Unclear	Low	Low	Low	High
Goettner 2005	Case control	High	High	Unclear	High	High	Low	High
Goossens 2000	Case control	High	High	Unclear	Low	Unclear	Low	High
Goossens 2001	Case control	High	High	Unclear	Low	Unclear	Low	High
Gueglio 1996	Case control	High	High	Unclear	Low	Unclear	Low	High
Hansen 1988	Case control	High	High	High	High	Unclear	Low	High
Hansen 1989	Case control	High	High	High	High	Low	Low	High
Hansen 1991	Case control	High	High	High	Low	Low	Low	High
Hernandez 2003	Case control	High	High	Unclear	Low	Unclear	Low	High
Hofmann 1990	Case control	High	High	Unclear	Low	Unclear	Low	High
Hofmann 1996	Case control	High	High	Unclear	Low	Unclear	Low	High
Hofstad 1987	Case control	High	High	High	High	Low	Low	High
Hunfeld 2002	Case control	High	High	Unclear	Low	Unclear	Low	High
Jovivic 2003	Case control	High	High	High/Unclear*	High	Unclear	Low	High
Kaiser 1998	Case control	High	High	High	High	High	Low	High
Kaiser 1999inf	Case control	High	High	High	High	High	Low	High
Karlsson 1989eur	Case control	High	High	High/Unclear*	High	Low	Low	High
Karlsson 1989siid	Case control	High	High	High	High	Low	Low	High
Lahdenne 2003	Case control	High	High	High	Low	Unclear	Low	High
Lakos 2005	Case control	High	High	Low	High/Low*	High	Low	High
Lange 1991	Case control	High	High	Unclear	High/Low*	Unclear	Low	High
Lencakova 2008	Case control	High	High	Low/Unclear*	High/Low*	Unclear	Low	High
Marangoni 2005jmm	Case control	High	High	Unclear	Low	Low	Low	High
Marangoni 2005new	Case control	High	High	Unclear	Low*	Low	Low	High
Marangoni 2008	Case control	High	High	Unclear	Unclear**	Unclear	Low	High
Mathiesen 1996	Case control	High	High	High/Low*	High/Low*	High	Low	High
Mathiesen 1998	Case control	High	High	High/Unclear*	High/Low*	Low	Low	High
Nicolini 1992	Case control	High	High	High	High	Unclear	Low	High
Nohlmans 1994	Case control	High	High	High/Unclear*	High/Low*	Unclear	Low	High
Oksi 1995	Case control	High	High	High/Unclear*	High/Low*	Unclear	Low	High
Olsson 1991	Case control	High	High	High	High	Unclear	Low	High
Panelius 2001	Case control	High	High	High	High	High	Low	High
Putzker 1995	Case control	High	High	Unclear	Low	High	Low	High
Rauer 1995	Case control	High	High	High	High	High	Low	High
Reiber 2013	Case control	High	High	High	Unclear	Low	Low	High
Rijpkema 1994	Case control	High	High	Unclear	High/Low*	Unclear	Low	High
Ruzic 2002	Case control	High	High	Unclear	Low	Unclear	Low	High
Ryffel 1998	Case control	High	High	Unclear	High	High	Low	High
Schulte 2004	Case control	High	High	Unclear	High	High	Low	High
Smismans 2006	Case control	High	High	Unclear	Low	High	Low	High
Tjernberg 2007	Case control	High	High	Unclear	Low/Unclear*	High	Low	High
Tjernberg 2011	Case control	High	High	Unclear	Low	High	Low	High
VanBurgel 2011	Case control	High	High	Unclear	Low	High	Low	High
Wilske 1993	Case control	High	High	High/Unclear*	High/Low*	High	Low	High
Wilske 1999	Case control	High	High	Unclear	High	High	Low	High
Zoller 1990	Case control	High	High	Unclear	High	Unclear	Low	High

RoB_P: Risk of Bias in patient sampling; RoB_I: Risk of Bias in Index test; RoB_RS: Risk of Bias in Reference Standard; RoB_TaF: Risk of Bias in timing and flow. CrA_P: Concerns regarding applicability of patient sample; CrA_I: Concerns regarding applicability of Index Test; CrA_RS: Concerns regarding applicability of Reference Standard. * some studies evaluated more than one test and evaluated these in different ways (e.g. for one test the cut-off value was pre-specified, while for the other test it was based on the results)

**Table 2:** Quality assessment of included cross-sectional control studies

Author_Year	Design	RoB_P	CrA_P	RoB_I	CrA_I	RoB_RS	CrA_RS	RoB_TaF
Albisetti 1997	Cross sectional	Low	Low	Unclear	Low	Low	Low	Unclear
Barrial 2011	Cross sectional	Unclear	Unclear	Low	Low	Low	Low	Low
Bazovska 2001	Cross sectional	Unclear	Unclear	Unclear	Low	Unclear	Low	Low
Bednarova 2006	Cross sectional	Unclear	Unclear	Unclear	Low	Unclear	Low	Unclear
Bennet 2008	Cross sectional	Unclear	High	Unclear	Low	Unclear	Low	Unclear
Blaauw 1993	Cross sectional	Low	Low	High	High	Low	Low	Low
Blaauw 1999	Cross sectional	Low	High	Unclear	High/Low*	Low	Low	Unclear
Blanc 2007	Cross sectional	Unclear	Unclear	Unclear	Low	High	Low	Unclear
Cermakova 2005	Cross sectional	Low	Low	Unclear	Low	High	Low	Low
Davidson 1999	Cross sectional	Unclear	Unclear	Unclear	High/Low*	High	High	Low
Ekerfelt 2004	Cross sectional	Unclear	Unclear	Low	Low	Unclear	Low	Unclear
Jansson 2005	Cross sectional	Unclear	Unclear	Unclear	Low	High	High	Low
Kolmel 1992	Cross sectional	Unclear	Unclear	Unclear	Low	High	Low	Unclear
Ljostad 2005	Cross sectional	High	High	Unclear	Unclear	Low	Low	Low
Popperl 2000	Cross sectional	Unclear	Unclear	Unclear	Low	High	High	Low
Roux 2007	Cross sectional	High	Unclear	Unclear	High/Low*	Low	High	Unclear
Skarpaas 2007	Cross sectional	Low	Low	High/Unclear*	Low	Unclear	Low	Unclear
Skogman 2008	Cross sectional	Unclear	Unclear	High	High	Low	Low	Low

RoB_P: Risk of Bias in patient sampling; RoB_I: Risk of Bias in Index test; RoB_RS: Risk of Bias in Reference Standard; RoB_TaF: Risk of Bias in timing and flow. CrA_P: Concerns regarding applicability of patient sample; CrA_I: Concerns regarding applicability of Index Test; CrA_RS: Concerns regarding applicability of Reference Standard. * some studies evaluated more than one test and evaluated these in different ways (e.g. for one test the cut-off value was pre-specified, while for the other test it was based on the results)

### Meta-analyses

#### Erythema migrans

Nineteen case-control studies including healthy controls evaluated the accuracy of serological tests for EM. The summary sensitivity for ELISA or immunoblot detecting EM patients was 50 % (95 % CI 40 % to 61 %) and specificity 95 % (95 % CI 92 % to 97 %). ELISA tests had a higher accuracy than immunoblots (*P*-value = 0 · 008), mainly due to a higher sensitivity (Table 3). Commercial tests did not perform significantly different from in-house tests. The 23 case-control studies on EM including cross-reacting controls had similar results (data not shown). One cross-sectional study done on EM-suspected patients evaluated four different immunoblots in patients with a positive or unclear ELISA result; their sensitivity varied between 33 and 92 % and their specificity between 27 and 70 % [66].

**Table 3:** Summary estimates of sensitivity and specificity for all case-definitions, derived from a hierarchical summary ROC model. The results may be different from those in the main text, as here they are specified for immunoblots and ELISAs and for commercial and in-house tests separately, while in the main text the overall estimates are provided

Case definition	Assay	Design	N (studies); N(2×2 tables); N(cases); N(controls)	Sensitivity (95 % CI)	Specificity (95 % CI)	Heterogeneity	Quality and Study Design
Erythema migrans	In-house ELISA	Case-control, Healthy controls	6, 10, 451, 658	0•41 (0•25 to 0•60)	0•97 (0•95 to 0•98)	IgG lower sensitivity than IgM. Other sources of heterogeneity were not found.	Study quality did not influence the accuracy
	In-house IB		3, 3, 182, 380	0•52 (0•38 to 0•65)	0•98 (0•94 to 0•99)		
	Commercial ELISA		13, 32, 874, 2509	0•54 (0•44 to 0•65)	0•93 (0•90 to 0•95)		
	Commercial IB		3, 5, 161, 289	0•58 (0•49 to 0•67)	0•86 (0•75 to 0•93)		
	Two-tiered tests		2, 7, 125, 190	range 0•12 to 0•64	range 0•67 to 0•96		
Lyme neuroborreliosis	In-house ELISA	Case-control, Healthy controls	6, 9, 277, 649	0•69 (0•60 to 0•76)	0•88 (0•72 to 0•97)	IgM and IgG have similar sensitivity and specificity, IgG has a higher accuracy. Recombinant tests perform best. More recent studies perform better than earlier studies.	If serology was not part of the reference standard, then specificity was lower.
(serum)	In-house IB		5, 8, 253, 445	0•69 (0•57 to 0•80)	0•93 (0•86 to 0•97)		
	Commercial ELISA		11, 28, 484, 2920	0•81 (0•70 to 0•89)	0•94 (0•91 to 0•96)		
	Commercial IB		2, 4, 33, 286	0•81 (0•57 to 0•94)	0•92 (0•88 to 0•95)		
	Two-tiered tests		1, 5, 15, 100	range 0•41 to 0•87	range 0•88 to 0•94		
(csf)	Any ELISA	Case-control, Cross-reacting controls	6, 9, 385, 261	0•74 (0•38 to 0•93)	0•96 (0•85 to 0•99)		
(serum + csf)	Specific AI test		7, 10, 458, 380	0•86 (0•63 to 0•95)	0•94 (0•85 to 0•97)		
Lyme neuroborreliosis	Any ELISA or IB (in serum)	Cross-sectional study	6, 12, 282, 412	0•78 (0•53 to 0•92)	0•78 (0•40 to 0•95)	Sensitivity similar for IgG and IgM; specifcity higher for IgG. No other sources of heterogeneity.	
	Specific AI test (in serum and CSF)		4, 4, 102, 118	0•79 (0•34 to 0•97)	0•96 (0•64 to 1•00)		
Lyme arthritis	All ELISA	Case-control, Healthy controls	8, 26, 160, 1112	Median0•96 Interquartile range 0•93 to 1•00	Median 0•94 Interquartile range 0•91 to 0•97	IgM a much lower sensitivity than IgG. No other sources of heterogeneity.	Study quality did not influence the accuracy
Acrodermatitis	All ELISA	Case-control, Healthy controls	10, 27, 256, 1415	0•97 (0•94 to 0•99)	0•95 (0•88 to 0•98)	IgM a much lower sensitivity than IgG. No other sources of heterogeneity.	Study quality did not influence the accuracy
Lyme borreliosis (unspecified)	In-house ELISA	Case-control, Healthy controls	4, 7, 115, 215	0•85 (0•71 to 0•93)	0•98 (0•93 to 0•99)	Tests assessing both IgM and IgG have highest sensitivity; specificity not very variable. Recombinant tests and more recent studies perform worse.	If serology was not part of the reference standard, then accuracy was lower.
	In-house IB		2, 4, 98, 126	0•63 (0•33 to 0•86)	0•97 (0•93 to 0•99)		
	Commercial ELISA		10, 43, 658, 815	0•70 (0•52 to 0•83)	0•95 (0•89 to 0•98)		
	Commercial IB		1, 4, 26, 62	0•29 (0•07 to 0•68)	0•96 (0•90 to 0•98)		
Lyme borreliosis (unspecified)	Any ELISA or IB	Cross-sectional study	5, 14, 226, 914	0•77 (0•48 to 0•93)	0•77 (0•46 to 0•93)	IgM lowest sensitivity, but highest specificity; no other sources investigated.	

Number of studies is for each combination of case definition and assay category. Thus the same study may appear more than once. *ELISA* Enzyme Immuno Assay, *IB* Immunoblot, *AI* Antibody Index, *CSF* Cerebrospinal Fluid

#### Neuroborreliosis

**Fig. 3:**
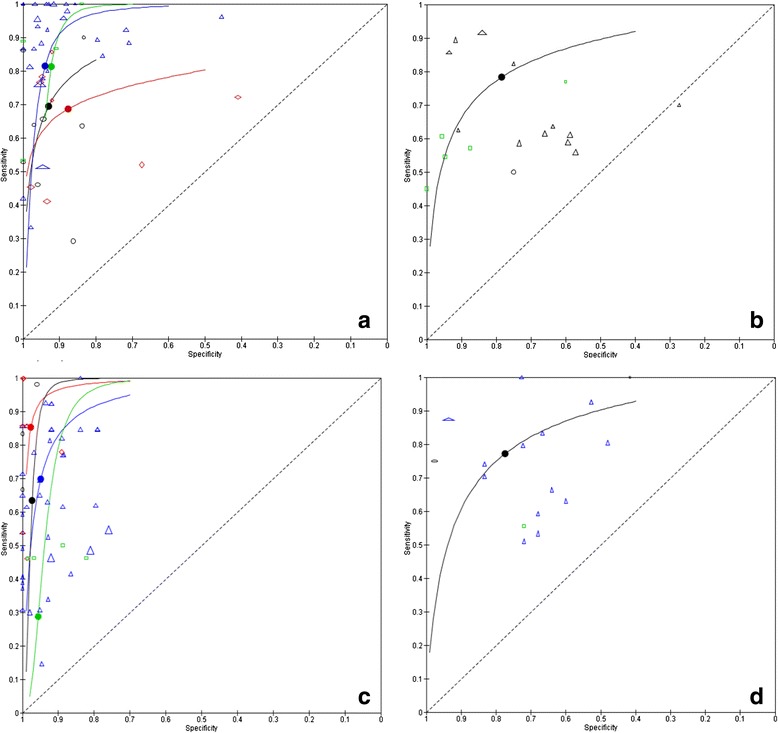
Raw ROC plots and fitted summary ROC curves. Every symbol reflects a 2 × 2 table, one for each test. Blue triangle = commercial EIA; Red diamond = in house EIA; Green rectangle = commercial IB; Black circle = in house IB. One study may have contributed more than one 2 × 2 table. The dots on the summary ROC curves reflect the summary estimate of sensitivity and specificity. **a** neuroborreliosis case-control studies including healthy controls. **b**: neuroborreliosis cross-sectional studies. **c** unspecified Lyme borreliosis case-control studies including healthy controls. **d** unspecified Lyme borreliosis cross-sectional studies. The size of the symbol reflects the sample size. For the cross-sectional studies, only the overall summary ROC curve is shown, while for the case-control designs the curves are shown for the different test-types

Twenty case-control studies on neuroborreliosis included healthy controls. Their overall sensitivity was 77 % (95 % CI 67 % to 85 %) and their specificity 93 % (95 % CI 88 % to 96 %) (Fig. 3a). On average, ELISA assays had a lower accuracy than immunoblot assays (*P* = 0 · 042). The in-house ELISAs had the lowest specificity of all tests (Table 3). Twenty-six case-control studies with cross-reacting controls showed similar results, but with a lower specificity (data not shown). The ten cross-sectional studies for neuroborreliosis had a median prevalence of 50 % (IQR 37 % to 70 %). The summary sensitivity for any serological test done in serum was 78 % (95 % CI 53 % to 92 %) and specificity was 78 % (95 % CI 40 % to 95 %) (Fig. 3b). Whether a test was ELISA or immunoblot, commercial or in-house did not affect model parameters.

#### Lyme Arthritis

Meta-analysis was not possible for the eight case-control studies on Lyme arthritis with healthy controls. We therefore only report the median estimates and their interquartile range (IQR). Median sensitivity was 96 % (IQR 93 % to 100 %); median specificity was 94 % (IQR 91 % to 97 %) (Table 3). Three cross-sectional studies were done in patients suspected of Lyme arthritis; this was insufficient to do a meta-analysis [66, 71, 85].

#### Acrodermatitis chronica atrophicans

The nine case control studies on ACA including a healthy control group had a high summary sensitivity for any serological assay: 98 % (95 % CI 84 % to 100 %). Specificity was 94 % (95 % CI 90 % to 97 %). One study had an extremely low sensitivity for the in-house assay evaluated; most likely because one of the antigens used (OspC) is no longer expressed by the spirochetes in long-standing disease [45]. Test-type was not added to the analyses, because of insufficient data. Case-control studies for ACA including cross-reacting controls had a lower sensitivity and specificity than the healthy control designs (both 91 %).

#### Unspecified Lyme borreliosis

Thirteen case-control studies included unspecified Lyme borreliosis cases and healthy controls. Their summary sensitivity for any test was 73 % (95 % CI 53 % to 87) and specificity was 96 % (95 % CI 91 % to 99 %) (Fig. 3c). Commercial tests had a lower accuracy (*P*-value = 0 · 008), mainly due to a lower sensitivity (Table 3). Twelve studies including cross-reacting controls had a summary sensitivity of 81 % (95 % CI 64 % to 91 %) and specificity of 90 % (95 % CI 79 % to 96 %). Five cross-sectional studies aimed to diagnose an unspecified form of Lyme borreliosis (Fig. 3d). The prevalence varied between 10 and 79 %, indicating a varying patient spectrum. Sensitivity was 77 % (95 % CI 48 % to 93 %) and specificity 77 % (95 % CI 46 % to 93 %). There were insufficient data points to analyze test type.

#### Two-tiered tests

One case-control study investigated the diagnostic accuracy of two-tiered approaches for all manifestations and healthy controls [11]. The sensitivity of the European algorithms varied between 55 % for EM and 100 % for ACA. The specificity for all assays was ≥ 99 %. Another case-control study investigated 12 different algorithms for ‘late Lyme borreliosis’ and ‘early Lyme borreliosis’ [21]. Their sensitivity varied between 4 and 50 % and the specificity varied between 88 and 100 %. One case-control study including EM cases and healthy controls and evaluating two algorithms reported a sensitivity of 11 % or 43 % and a specificity of 100 % [14]. Two cross-sectional studies on two-tiered tests aimed at diagnosing neuroborreliosis [80, 81] and two at diagnosing unspecified Lyme borreliosis [67, 70]. Their prevalence varied between 19 and 77 %; their sensitivity between 46 and 97 %; and their specificity between 56 and 100 %.

#### Specific antibody index

Seven studies containing cross-reacting controls evaluated a specific antibody index for the diagnosis of neuroborreliosis. The summary sensitivity was 86 % (95 % CI 63 % to 95 %) and specificity 94 % (95 % CI 85 % to 97 %). The four cross-sectional studies had a summary sensitivity of 79 % (95 % CI 34 % to 97 %) and a summary specificity of 96 % (95 % CI 64 % to 100 %).

#### Heterogeneity

The IgG tests had a comparable sensitivity to the IgM tests, except for EM (IgM slightly higher sensitivity), Lyme arthritis and ACA (IgM much lower sensitivity in both). Tests assessing both IgM and IgG had the highest sensitivity and the lowest specificity, although specificity was above 80 % in most cases. (Table 4).

**Table 4:** Summary estimates of sensitivity and specificity for IgM versus IgG versus IGM or IgG (IgT)

		IgM	IgG	IgT
		Estimate (95 % CI)	Estimate (95 % CI)	Estimate (95 % CI)
Erythema migrans	Sensitivity	0.426 (0.361 to 0.494)	0.359 (0.293 to 0.431)	0.606 (0.503 to 0.700)
	Specifcitiy	0.953 (0.924 to 0.971)	0.961 (0.939 to 0.975)	0.919 (0.885 to 0.944)
Neuroborreliosis	Sensitivity	0.600 (0.526 to 0.669)	0.589 (0.515 to 0.659)	0.865 (0.812 to 0.906)
	Specifcitiy	0.949 (0.924 to 0.966)	0.956 (0.935 to 0.971)	0.913 (0.869 to 0.942)
Lyme arthritis	Sensitivity	0.392 (0.279 to 0.517)	0.941 (0.857 to 0.977)	0.945 (0.842 to 0.982)
	Specifcitiy	0.951 (0.881 to 0.980)	0.969 (0.942 to 0.983)	0.921 (0.837 to 0.964)
Acrodermatitis Chronica Atrophicans	Sensitivity	0.184 (0.090 to 0.340)	0.987 (0.821 to 0.999)	0.978 (0.874 to 0.996)
	Specifcitiy	0.965 (0.930 to 0.983)	0.966 (0.952 to 0.976)	0.932 (0.883 to 0.962)
Unspecified Lyme borreliosis*	Sensitivity	0.596 (0.324 to 0.820)	0.557 (0.448 to 0.661)	0.792 (0.960 to 0.867)
	Specifcitiy	0.911 (0.818 to 0.959)	0.986 (0.877 to 0.998)	0.947 (0.725 to 0.992)

IgT refers to assays measuring IgM and IgG simultaneously. 95 % CI = 95 % confidence interval. *Analyses were not possible for healthy controls; these are the estimates for studies including cross-reacting controls

We evaluated the effect of three antigen types: whole cell, purified proteins or recombinant antigens. In neuroborreliosis, recombinant antigens had both the highest sensitivity and specificity, while in unspecified Lyme, they had the lowest sensitivity and specificity. (Table 5) Year of publication showed an effect only for erythema migrans and neuroborreliosis: in both cases publications before the year 2000 showed a lower sensitivity than those after 2000. (Table 6) Antigen type and year of publication were not associated with each other.

**Table 5:** Generation of antigens

	Antigen	Sensitivity (95 % CI)	Specificity (95 % CI)
Erythema migrans	Whole cell	0.515 (0.328 to 0.699)	0.957 (0.899 to 0.983)
	Purified	0.579 (0.466 to 0.685)	0.950 (0.895 to 0.977)
	Recombinant	0.551 (0.330 to 0.753)	0.947 (0.881 to 0.977)
Neuroborreliosis	Whole cell	0.723 (0.555 to 0.845)	0.904 (0.792 to 0.959)
	Purified	0.756 (0.614 to 0.858)	0.963 (0.935 to 0.979)
	Recombinant	0.837 (0.647 to 0.935)	0.931 (0.881 to 0.960)
Lyme arthritis*	Whole cell or Purified	0.952 (0.892 to 0.979)	0.958 (0.879 to 0.986)
	Recombinant	0.954 (0.862 to 0.985)	0.927 (0.886 to 0.954)
Unspecified Lyme borreliosis	Whole cell	0.703 (0.563 to 0.813)	0.950 (0.863 to 0.983)
	Purified	0.836 (0.463 to 0.968)	0.965 (0.855 to 0.992)
	Recombinant	0.464 (0.251 to 0.692)	0.918 (0.806 to 0.968)

For ACA there were insufficient data to analyse the effect of antigen used, so ACA is not in the table. 95 % CI = 95 % confidence interval. *Insufficient data to analyse whole cell assays and purified antigen assays separately

**Table 6:** Year of publication

	Year of publication	Sensitivity (95 % CI)	Specificity (95 % CI)
Erythema migrans	<2000	0.631 (0.515 to 0.733)	0.897 (0.818 to 0.944)
	2000 or later	0.853 (0.724 to 0.928)	0.929 (0.826 to 0.973)
Neuroborreliosis	<2000	0.631 (0.515 to 0.733)	0.897 (0.818 to 0.944)
	2000 or later	0.853 (0.724 to 0.928)	0.929 (0.826 to 0.973)

95 % CI = 95 % confidence interval

For unspecified Lyme we were able to directly compare the accuracy in early stages of disease with the accuracy in later stages of disease. The tests showed a lower sensitivity and slightly higher specificity in the early stages of the disease. (Table 7).

**Table 7:** Early versus late Lyme borreliosis

		Overall	Early Lyme	Late Lyme
		Estimate (95 % CI)	Estimate (95 % CI)	Estimate (95 % CI)
Unspecified Lyme borreliosis	Sensitivity	0.774 (0.468 to 0.930)	0.600 (0.323 to 0.826)	0.798 (0.554 to 0.926)
	Specifcitiy	0.960 (0.852 to 0.990)	0.968 (0.904 to 0.990)	0.957 (0.875 to 0.986)

95 % CI = 95 % confidence interval

We were able to meta-analyze manufacturer-specific results for only two manufacturers, but the results showed much variability and the confidence intervals were broad.

We investigated the effect of the reference standard domain of QUADAS-2: acceptable case definition versus no or unclear; and serology in the case definition versus no or unclear. None had a significant effect on accuracy. The study by Ang contained at least 8 different 2x2 tables for each case definition and may have therefore weighed heavily on the results [8]. However, sensitivity analysis showed that its effect was only marginal. The same was true for assuming possible cases as being controls and indeterminate test results as being negatives.

## Discussion

Overall, the diagnostic accuracy of ELISAs and immunoblots for Lyme borreliosis in Europe varies widely, with an average sensitivity of ~80 % and a specificity of ~95 %. For Lyme arthritis and ACA the sensitivity was around 95 %. For EM the sensitivity was ~50 %. In cross-sectional studies of neuroborreliosis and unspecified Lyme borreliosis, the sensitivity was comparable to the case-control designs, but the specificity decreased to 78 and 77 % respectively. Two-tiered tests did not outperform single tests. Specific antibody index tests did not outperform the other tests for neuroborreliosis, although the specificity remained high even in the cross-sectional designs. All results should be interpreted with caution, as the results showed much variation and the included studies were at high risk of bias.

Although predictive values could not be meta-analyzed, the sensitivity and specificity estimates from this review may be used to provide an idea of the consequences of testing when the test is being used in practice. Imagine that a clinician sees about 1000 people a year who are suspected of one of the manifestations of Lyme borreliosis, in a setting where the expected prevalence of that manifestation is 10 %. A prevalence of 10 % would mean that 100 out of 1000 tested patients will really have a form of Lyme borreliosis. If these people are tested by an ELISA with a sensitivity 80 %, then 0.80*100 = 80 patients with Lyme borreliosis will test positive and 20 patients will test negative. If we assume a specificity of 80 % as well (following the estimates from the cross-sectional designs), then out of the 900 patients without Lyme borreliosis, 0.80*900 = 720 will test negative and 180 will test positive. These numbers mean that in this hypothetical cohort of 1000 tested patients, 80 + 180 = 260 patients will have a positive test result. Only 80 of these will be true positives and indeed have Lyme borreliosis (positive predictive value 80/260 = 0.31 = 31 %). The other 180 positively tested patients are the false positives and they will be treated for Lyme while they have another cause of disease, thus delaying their final diagnosis and subsequent treatment. In a two-tiered approach, all positives will be tested with immunoblot after ELISA. These numbers also mean that we will have 720 + 20 = 740 negative test results, of which 3 % (negative predictive value 720/740 = 0.97 = 97 %) will have Lyme borreliosis despite a negative test result. These are the false-negatives, their diagnosis will be missed or delayed. Although calculations like these may provide insight in the consequences of testing, they should be taken with caution. The results were overall very heterogeneous and may depend on patient characteristics. Also, the prevalence of 10 % may not be realistic. In our review, we found prevalences ranging from 1 to 79 % for unspecified Lyme borreliosis and ranging from 12 to 62 % for neuroborreliosis. Appendix 3 shows some more of these inferences, for different prevalence situations and different sensitivity and specificity of the tests.

Limitations of this review are the representativeness of the results, the poor reporting of study characteristics and the lack of a true gold standard. Most studies included were case-control studies. These may be easier to perform in a laboratory setting than cross-sectional designs, but their results are less representative for clinical practice. Also the immunoblot was not analysed in a way that is representative for practice: most immunoblots were analysed on the same samples as the ELISAs, while in practice immunoblots will only be used on ELISA-positive samples. EM patients formed the second largest group of patients in our review. The low sensitivity in this group of patients supports the guidelines stating that serological testing in EM patients is not recommended [86]. On the other hand, patients with atypical manifestations were not included in the reviewed studies, while this group of patients does form a diagnostic problem [87, 88]. A more detailed analyses of the included patients’ characteristics and test characteristics would have been nice, but these characteristics were poorly reported. This is also reflected in the Quality-assessment table, with many ‘unclear’ scores, even for more recent studies. Authors may not have been aware of existing reporting guidelines and we therefore suggest that authors of future studies use the STAndards for Reporting Diagnostic accuracy studies (STARD) to guide their manuscript [89].

There is no gold standard for Lyme borreliosis, so we used the reference standard as presented by the authors of the included studies. This may have added to the amount of variation. Furthermore, many of the investigated studies included the results from antibody testing in their definition of Lyme borreliosis, which may have overestimated sensitivity and specificity. However, this was not proven in our heterogeneity analyses.

The performance of diagnostic tests very much depends on the population in which the test is being used. Future studies should therefore be prospective cross-sectional studies including a consecutive sample of presenting patients, preferably stratified by the situation in which the patient presents (e.g. a tertiary Lyme referral center versus general practice). The lack of a gold standard may be solved by using a reference standard with multiple levels of certainty [90, 91]. Although this will diminish contrasts and will thus be more difficult to interpret, it does reflect practice in a better way. Other solutions may be more statistically derived approaches like latent class analysis, use of expert-opinion and/or response to treatment [92].

However, more and better designed diagnostic accuracy studies will not improve the accuracy of these tests themselves. They will provide more valid estimates of the tests’ accuracy, including predictive values, but the actual added value of testing for Lyme disease requires information about subsequent actions and consequences of testing. If the final diagnosis or referral pattern is solely based upon the clinical picture, then testing patients for Lyme may have no added value. In that case, a perfect test may still be useless if it does not change clinical management decisions [93]. On the other hand, imperfect laboratory tests may still be valuable for clinical decision making if subsequent actions improve the patient’s outcomes. The challenge for clinicians is to deal with the uncertainties of imperfect laboratory tests.

## Conclusions

We found no evidence that ELISAs have a higher or lower accuracy than immunoblots; neither did we find evidence that two-tiered approaches have a better performance than single tests. However, the data in this review do not provide sufficient evidence to make inferences about the value of the tests for clinical practice. Valid estimates of sensitivity and specificity for the tests as used in practice require well-designed cross-sectional studies, done in the relevant clinical patient populations. Furthermore, information is needed about the prevalence of Lyme borreliosis among those tested for it and the clinical consequences of a negative or positive test result. The latter depend on the place of the test in the clinical pathway and the clinical decisions that are driven by the test results or not. Future research should primarily focus on more targeted clinical validations of these tests and research into appropriate use of these tests.

## Availability of data and materials

The raw data (data-extraction results, reference lists, statistical codes) will be provided upon request by ECDC (info@ecdc.europa.eu).

## Appendix 1

**Table 8:** Search strategy

Search strategy in Ovidsp: database Embase 1980-present date search January 10, 2013; for the update in February 2014 we used the same strategy		
Line#	Term	Results
1	exp serology/	171111
2	serolog*.ti,ab,ot.	99141
3	antibod*.ti,ab,ot.	739175
4	exp antibody/	745964
5	immunoglobin*.ti,ab,ot.	1034
6	IgG.ti,ab,ot.	119346
7	IgM.ti,ab,ot.	58826
8	exp enzyme linked immunosorbent assay/	185612
9	ELISA.ti,ab,ot.	136569
10	exp immunoassay/	343973
11	EIA.ti,ab,ot.	10028
12	immunosorbent.ti,ab,ot.	64523
13	immunofluorescent.ti,ab,ot.	16475
14	immunofluorescence.ti,ab,ot.	87513
15	immunoblot*.ti,ab,ot.	67674
16	“western blot”.ti,ab,ot.	98174
17	immunoassay.ti,ab,ot.	45568
18	exp lymphocyte transformation test/	1200
19	“lymphocyte transformation test”.ti,ab,ot.	826
20	LTT.ti,ab,ot.	574
21	((t-cell* or lymphocyte) adj15 (diagnostic or diagnosis or diagnosing or screen* or test*)).mp.	41706
22	VIDAS.ti,ab,ot.	709
23	liason.ti,ab,ot.	104
24	Enzygnost.ti,ab,ot.	233
25	Serion.ti,ab,ot.	58
26	recomline.ti,ab,ot.	21
27	(virotech adj5 (europline or “line blot”)).ti,ab,ot.	1
28	euroimmunoblot.ti,ab,ot.	0
29	diacheck.ti,ab,ot.	2
30	euroimmun.ti,ab,ot.	191
31	Medac.ti,ab,ot.	136
32	mikrogen$.ti,ab,ot.	78
33	virotech.ti,ab,ot.	53
34	ELISPOT.ti,ab,ot.	5408
35	exp enzyme linked immunospot assay/	4884
36	(c6 adj3 immunetics).ti,ab,ot.	5
37	or/1-36	1629954
38	lyme.ti,ab,ot.	10067
39	Lyme borreliosis/	11571
40	tick borne disease/	1803
41	exp tick bite/	1956
42	(tick adj2 bite).ti,ab,ot.	1400
43	Neuroberreliosis.ti,ab,ot.	0
44	exp erythema chronicum migrans/	1641
45	erythema migrans.ti,ab,ot.	1187
46	“erythema chronicum migrans”.ti,ab,ot.	433
47	exp Acrodermatitis chronica atrophicans/	155
48	“Acrodermatitis chronica atrophicans”.ti,ab,ot.	426
49	meningoradiculitis.ti,ab,ot.	269
50	lyme.ti,ab,ot.	10067
51	Lyme borreliosis/	11571
52	tick borne disease/	1803
53	exp tick bite/	1956
54	(tick adj2 bite).ti,ab,ot.	1400
55	Neuroborreliosis.ti,ab,ot.	1091
56	exp erythema chronicum migrans/	1641
57	erythema migrans.ti,ab,ot.	1187
58	“erythema chronicum migrans”.ti,ab,ot.	433
59	exp Acrodermatitis chronica atrophicans/	155
60	“Acrodermatitis chronica atrophicans”.ti,ab,ot.	426
61	meningoradiculitis.ti,ab,ot.	269
62	lyme.ti,ab,ot.	10067
63	Lyme borreliosis/	11571
64	tick borne disease/	1803
65	exp tick bite/	1956
66	(tick adj2 bite).ti,ab,ot.	1400
67	exp erythema chronicum migrans/	1641
68	erythema migrans.ti,ab,ot.	1187
69	“erythema chronicum migrans”.ti,ab,ot.	433
70	exp Acrodermatitis chronica atrophicans/	155
71	“Acrodermatitis chronica atrophicans”.ti,ab,ot.	426
72	meningoradiculitis.ti,ab,ot.	269
73	Neuroborreliosis.ti,ab,ot.	1091
74	or/38-73	16588
75	exp Borrelia/	10262
76	borrelia.ti,ab,ot.	8824
77	burgdorferi.ti,ab,ot.	7378
78	Borrelia infection/	2668
79	or/75-78	12748
80	VLsE.ti,ab,ot.	142
81	OspC.ti,ab,ot.	446
82	or/80-81	551
83	74 or 79	20529
84	82 or 83	20551
85	37 and 84	7578
86	37 or 82	1630187
87	83 and 86	7789
88	85 or 87	7790
89	animal/not human/	1348171
90	88 not 89	7369
91	review.pt.	1907269
92	90 not 91	6510

## Appendix 2 Data quality

### Patient selection

#### Risk of bias, signalling questions

- Was a case-control design avoided?
  - ○ Case-control designs, especially if they include healthy controls, possess a high risk of bias. Therefore, all case-control studies are automatically judged to be of high risk of bias in the overall judgment.
- Was a consecutive or random sample of patients enrolled?
- Did the study avoid inappropriate exclusions?
- Overall judgment:
  - ○ Case-control studies always scored as high risk of bias
  - ○ Cross-sectional studies only low risk of bias if other two signalling questions are scored as ‘yes’. If one of them is scored ‘no’, then high risk of bias. Otherwise unclear.

#### Concerns regarding applicability

This is about the extent to which the patients (both cases and controls) included in this study are representative for the patients in which these serology tests will be used.

- Is there concern that the included patients do not match the review question?
  - ○ All case-control studies automatically high concern. All cross-sectional studies automatically low concern, except when no clear case-definition has been used.
  - ○ One study only included facial palsy patients → high concern: applicable, but not representative group
  - ○ One study only included arthritic patients → high concern: applicable, but not representative group

### Index test

#### Risk of bias, signalling questions

- Were the index test results interpreted without knowledge of the results of the reference standard?
- If a threshold was used, was it pre-specified? By selecting the cut-off value with the highest sensitivity and/or specificity, researchers artificially optimize the accuracy of their tests, which also may cause overestimation of sensitivity and specificity.
  - ○ The first question is very poorly reported, almost in all cases ‘unclear’.
  - ○ The second question varies. Sometimes the authors state that 95% value of the controls is used as threshold, or that the mean of the controls plus 2 or 3 SD is used as threshold. Both variation have been scored as post-hoc.
- Overall judgment:
  - ○ if the second question is scored as ‘yes’, then automatically overall judgement also scored as yes. This is because the first question will usually be not reported or scored as ‘yes’.
  - ○ If the latter is scored as unclear, then overall also unclear; if latter is scored as no, then overall high risk.

#### Concerns regarding applicability

This is about the extent to which the index test evaluated is representative for the tests that will be used in practice.

- Is there concern that the index test, its conduct or interpretation differ from the review question?
  - ○ All in-house tests automatically scored as high concern

Risk of bias and concerns regarding applicability should be scored for each test separately.

### Reference Standard

#### Risk of bias, signalling questions

- Is the reference standard likely to correctly classify the target condition?
  - ○ Assumption: this will be likely in case-control studies that used the ‘correct’ case-definitions (e.g. Stanek [1], WHO)
  - ○ For cross-sectional studies also likely for studies that used the ‘correct’ case-definitions.
  - ○ Studies using Western blot as reference standard will be scored ‘no’ for this question.
- Were the reference standard results interpreted without knowledge of the results of the index test?
  - ○ Assumption: this will be likely in most case-control studies, but only if serology was not part of the case definition.
  - ○ For the cross-sectional studies, this should be explicitly stated.
- Overall judgement risk of bias:
  - ○ case control studies with clear case-definitions scored with low risk of bias;
  - ○ case control studies with unclear/wrong case-definitions score as unclear? Or high risk of bias?
  - ○ Cross-sectional studies with a clear case-definition and the second question scored as ‘yes’: low risk of bias.
  - ○ Otherwise unclear, as latter question will be very poorly reported?

#### Concerns regarding applicability

Is there concern that the target condition as defined by the reference standard does not match the review question?

- Western blot measures antibody response, while we are interested in Lyme borreliosis, irrespective of antibody status. So western blots are considered to have high concerns regarding applicability.
- If serology included in case definition, then incorporation bias and thus high risk of bias.
- If a case-control study used clear criteria and did not include serology in these criteria, then Low concern.

### Risk of bias regarding flow and timing, signalling questions

- Was there an appropriate interval between index test(s) and reference standard?
  - ○ We expected that in the cross-sectional designs most tests would have been done around the same moment as the final diagnosis was being made. If we suspect the patient status may have changed between the time of testing and the moment of diagnosis, then we scored this as ‘no’.
  - ○ For case-control studies this was always scored as ‘no’, as the determination of serology was always done after the case-definitions were defined, sometimes a long time after that was done.
- Did patients receive the same reference standard?
  - ○ Were scored as ‘no’ for all case-control studies, as the controls were often from different settings, different departments and had to fulfil different criteria.
- Were all patients included in the analysis?
  - ○ This was also scored ‘no’ for all case-control studies.
- Overall judgment:
  - ○ Case-control studies always scored high risk of bias
  - ○ For cross-sectional studies, we scored low risk of bias if all three questions were scored ‘yes’ and high risk of bias if at least one of them was scored ‘no’. All other cases were scored ‘unclear’.

## Appendix 3 Possible ranges in post-test probability

The Tables A to D show the absolute numbers of true positives, false positives, false negatives and true negatives for a hypothetical cohort of 1000 patients.

These numbers should be taken with caution, as the results were overall very heterogeneous. Furthermore, although the prevalence does not influence estimates of sensitivity and specificity extensively in our calculation, this assumption requires further elaboration.

To take into account variation in results and uncertainty, the calculations are made for different scenarios and presented in Tables A to D.

**Table:**

**TABLE A: specificity=95 %, prevalence=10 %**						**TABLE B: specificity=80 %, prevalence=10 %**				
Sensitivity:	TP	FP	FN	TN		Sensitivity:	TP	FP	FN	TN
0.90	90	45	10	855		0.90	90	180	10	720
0.85	85	45	15	855		0.85	85	180	15	720
0.80	80	45	20	855		0.80	80	180	20	720
0.75	75	45	25	855		0.75	75	180	25	720
0.70	70	45	30	855		0.70	70	180	30	720
0.60	60	45	40	855		0.60	60	180	40	720
0.50	50	45	50	855		0.50	50	180	50	720
**TABLE C: sensitivity=80 %, prevalence =10 %**						**TABLE D: sensitivity=80 %, specificity=80 %/95 %**				
Specificity:	TP	FP	FN	TN		Specificity:	0.95			
0.99	80	9	20	891		Prevalence	TP	FP	FN	TN
0.95	80	45	20	855		0.05	40	48	10	903
0.90	80	90	20	810		0.10	80	45	20	855
0.85	80	135	20	765		0.20	160	40	40	760
0.80	80	180	20	720		Specificity:	0.80			
0.75	80	225	20	675		Prevalence	TP	FP	FN	TN
0.70	80	270	20	630		0.05	40	190	10	760
0.65	80	315	20	585		0.10	80	180	20	720
0.60	80	360	20	540		0.20	160	160	40	640

Table A: varying sensitivities, at a fixed specificity of 95 % and a fixed prevalence of 10 %; Table B: varying sensitivities, at a more realistic fixed specificity of 80 % and a fixed prevalence of 10 %; Table C: varying specificities, at a fixed sensitivity of 80 % and a fixed prevalence of 10 %; Table D: sensitivity of 80 % and a specificity of 80 % and 95 %, at varying prevalence.

## Abbreviations

ACA: acrodermatitis chronica atrophicans
ELISA: enzyme-linked immunosorbent assays
EM: erythema migrans
HSROC: hierarchical summary ROC
Ig: immunoglobulin
QUADAS: QUAlity of Diagnostic Accuracy Studies
ROC: receiver operating characteristic

## References

[1] Hubálek Z (2009). Epidemiology of Lyme borreliosis. Curr Probl Dermatol.

[2] Stanek G, Wormser GP, Gray J, Strle F (2012). Lyme borreliosis. Lancet.

[3] Mulherin SA, Miller WC (2002). Spectrum bias or spectrum effect? Subgroup variation in diagnostic test evaluation. Ann Intern Med.

[4] Knottnerus JA, Muris JW (2003). Assessment of the accuracy of diagnostic tests: the cross-sectional study. J Clin Epidemiol.

[5] Rutjes AW, Reitsma JB, Vandenbroucke JP, Glas AS, Bossuyt PM (2005). Case-control and two-gate designs in diagnostic accuracy studies. Clin Chem.

[6] Whiting PF, Rutjes AW, Westwood ME (2011). QUADAS-2: a revised tool for the quality assessment of diagnostic accuracy studies. Ann Intern Med.

[7] Macaskill P, Gatsonis C, Deeks JJ, Harbord RM, Takwoingi Y. Chapter 10:Analysing and Presenting Results. In: Deeks JJ, Bossuyt PM, Gatsonis C, editors. Cochrane Handbook for Systematic Reviews of Diagnostic Test Accuracy Version 1.0. The Cochrane Collaboration, 2010. Available from: http://methods.cochrane.org/sdt/handbook-dta-reviews/.

[8] Ang CW, Brandenburg AH, van Burgel ND, Bijlmer HA, Herremans T, Stelma FF (2012). Nationale vergelijking van serologische assays voor het aantonen van Borrelia-antistoffen. Nederlands tijdschrift voor Medische Microbiologie.

[9] Ang CW, Notermans DW, Hommes M, Simoons-Smit AM, Herremans T (2011). Large differences between test strategies for the detection of anti-Borrelia antibodies are revealed by comparing eight ELISAs and five immunoblots. Eur J Clin Microbiol Inf Dis.

[10] Bergstrom S, Sjostedt A, Dotevall L, Kaijser B, Ekstrand-Hammarstrom B, Wallberg C (1991). Diagnosis of Lyme borreliosis by an enzyme immunoassay detecting immunoglobulin G reactive to purified Borrelia burgdorferi cell components. Eur J Clin Microbiol Inf Dis.

[11] Branda JA, Strle F, Strle K, Sikand N, Ferraro MJ, Steere AC (2013). Performance of United States serologic assays in the diagnosis of Lyme borreliosis acquired in Europe. Clin Infect Dis.

[12] Cerar T, Ogrinc K, Strle F, Ruzic-Sabljic E (2010). Humoral immune responses in patients with lyme neuroborreliosis. Clin Vaccine Immunol.

[13] Cerar T, Ruzic-Sabljic E, Cimperman J, Strle F (2006). Comparison of immunofluorescence assay (IFA) and LIAISON in patients with different clinical manifestations of Lyme borreliosis. Wien Klin Wochenschr.

[14] Christova I (2003). Enzyme-linked immunosorbent assay, immunofluorescent assay X and recombinant immunoblotting in the serodiagnosis of early Lyme borreliosis. Int J Imm Pharmacol.

[15] Cinco M, Murgia R (2006). Evaluation of the C6 enzyme-linked immunoadsorbent assay for the serodiagnosis of Lyme borreliosis in north-eastern Italy. New Microbiol.

[16] Dessau RB, Ejlertsen T, Hilden J (2010). Simultaneous use of serum IgG and IgM for risk scoring of suspected early Lyme borreliosis: Graphical and bivariate analyses. APMIS.

[17] Dessau RB (2013). Diagnostic accuracy and comparison of two assays for Borrelia-specific IgG and IgM antibodies: Proposals for statistical evaluation methods, cut-off values and standardization. J Med Microbiol.

[18] Flisiak R, Kalinowska A, Bobrowska E, Prokopowicz D (1996). Enzyme immunoassay in the diagnosis of Lyme borreliosis. Roczniki Akademii Medycznej w Bialymstoku.

[19] Flisiak R, Wierzbicka I, Prokopowicz D (1998). Western blot banding pattern in early Lyme borreliosis among patients from an endemic region of north-eastern Poland. Roczniki Akademii Medycznej w Bialymstoku.

[20] Goettner G, Schulte-Spechtel U, Hillermann R, Liegl G, Wilske B, Fingerle V (2005). Improvement of lyme borreliosis serodiagnosis by a newly developed recombinant immunoglobulin G (IgG) and IgM line immunoblot assay and addition of VlsE and DbpA homologues. J Clin Microbiol.

[21] Goossens HAT, van de Bogaard AE, Nohlmans MKE (1999). Evaluation of fifteen commercially available serological tests for diagnosis of Lyme borreliosis. Eur J Clin Microbiol Inf Dis.

[22] Goossens HAT, Van den Bogaard AE, Nohlmans MKE (2001). Serodiagnosis of Lyme borreliosis using detection of different immunoglobulin (sub)classes by enzyme-linked immunosorbent assay and Western blotting. Clin Lab.

[23] Gueglio B, Poinsignon Y, Berthelot JM, Marjolet M (1996). Borreliose De Lyme: Specificite De Western Blot. Med Mal Infect.

[24] Hansen K, Asbrink E (1989). Serodiagnosis of erythema migrans and acrodermatitis chronica atrophicans by the Borrelia burgdorferi flagellum enzyme-linked immunosorbent assay. J Clin Microbiol.

[25] Hansen K, Hindersson P, Pedersen NS (1988). Measurement of antibodies to the Borrelia burgdorferi flagellum improves serodiagnosis in Lyme borreliosis. J Clin Microbiol.

[26] Hansen K, Lebech AM (1991). Lyme neuroborreliosis: A new sensitive diagnostic assay for intrathecal synthesis of Borrelia burgdorferi-specific immunoglobulin G, A, and M. Ann Neurol.

[27] Hernandez-Novoa B, Orduna A, Bratos MA, Eiros JM, Fernandez JM, Gutierrez MP (2003). Utility of a commercial immunoblot kit (BAG-Borrelia blot) in the diagnosis of the preliminary stages of Lyme borreliosis. Diagn Microbiol Inf Dis.

[28] Hofmann H (1996). Lyme borreliosis - Problems of serological diagnosis. Infection.

[29] Hofmann H, Meyer-Konig U (1990). Serodiagnostik Bei Dermatologischen Krankheitsbildern Der Borrelia Burgdorferi-Infektion. Hautarzt.

[30] Hofstad H, Matre R, Myland H, Ulvestad E (1987). Bannwarth’s syndrome: Serum and CSF IgG antibodies against Borrelia burgdorferi examined by ELISA. Acta Neurol Scand.

[31] Hunfeld KP, Ernst M, Zachary P, Jaulhac B, Sonneborn HH, Brade V (2002). Development and laboratory evaluation of a new recombinant ELISA for the serodiagnosis of Lyme borreliosis. Wien Klin Wochenschr.

[32] Jovicic VL, Grego EM, Lako BL, Ristovic BM, Lepsanovic ZA, Stajkovic NT (2003). Improved serodiagnosis of early Lyme borreliosis: Immunoblot with local Borrelia afzelli strain. APMIS.

[33] Kaiser R, Rauer S (1998). Analysis of the intrathecal immune response in neuroborreliosis to a sonicate antigen and three recombinant antigens of Borrelia burgdorferi sensu stricto. Eur J Clin Microbiol Inf Dis.

[34] Kaiser R, Rauer S (1999). Serodiagnosis of neuroborreliosis: Comparison of reliability of three confirmatory assays. Infection.

[35] Karlsson M, Granstrom M (1989). An IgM-antibody capture enzyme immunoassay for serodiagnosis of Lyme borreliosis. Serodiagn Immunother Infect Dis.

[36] Karlsson M, Mollegard I, Stiernstedt G, Wretlind B (1989). Comparison of Western blot and enzyme-linked immunosorbent assay for diagnosis of Lyme borreliosis. Eur J Clin Microbiol Inf Dis.

[37] Lahdenne P, Panelius J, Saxen H, Heikkila T, Sillanpaa H, Peltomaa M (2003). Improved serodiagnosis of erythema migrans using novel recombinant borrelial BBK32 antigens. J Med Microbiol.

[38] Lakos A, Ferenczi E, Komoly S, Granström M (2005). Different B-cell populations are responsible for the peripheral and intrathecal antibody production inneuroborreliosis. Int Immunol.

[39] Lange R, Schneider T, Topel U, Mater-Bohm H, Beck A, Kolmel HW (1991). Borrelia burgdorferi antibody-tests: Comparison of IFT. ELISA Western-blot Lab Med.

[40] Lencakova D, Fingerle V, Stefancikova A, Schulte-Spechtel U, Petko B, Schreter I (2008). Evaluation of recombinant line immunoblot for detection of Lyme borreliosis in Slovakia: Comparison with two other immunoassays. Vector-Borne Zoonot.

[41] Marangoni A, Moroni A, Accardo S, Cevenini R (2008). Borrelia burgdorferi VlsE antigen for the serological diagnosis of Lyme borreliosis. Eur J Clin Microbiol Inf Dis.

[42] Marangoni A, Sparacino M, Cavrini F, Storni E, Mondardini V, Sambri V (2005). Comparative evaluation of three different ELISA methods for the diagnosis of early culture-confirmed Lyme borreliosis in Italy. J Med Microbiol.

[43] Marangoni A, Sparacino M, Mondardini V, Cavrini F, Storni E, Donati M (2005). Comparative evaluation of two enzyme linked immunosorbent assay methods and three Western Blot methods for the diagnosis of culture-confirmed early Lyme Borreliosis in Italy. New Microbiol.

[44] Mathiesen MJ, Christiansen M, Hansen K, Holm A, Asbrink E, Theisen M (1998). Peptide-based OspC enzyme-linked immunosorbent assay for serodiagnosis of Lyme borreliosis. J Clin Microbiol.

[45] Mathiesen MJ, Hansen K, Axelsen N, Halkier-Sorensen L, Theisen M (1996). Analysis of the human antibody response to outer surface protein C (OspC) of Borrelia burgdorferi sensu stricto, B. garinii, and B. afzelii. Med Microbiol Immunol.

[46] Nicolini P, Imbs P, Jaulhac B, Piemont Y, Warter JM, Monteil H (1992). Comparaison De L’immunofluorescence Indirecte Et Du Western Blot Pour Le Serodiagnostic Des Infections Neurologiques a Borrelia Burgdorferi. Med Mal Inf.

[47] Nohlmans MKE, Blaauw AAM, Van den Bogaard AEJ, Van Boven CPA (1994). Evaluation of nine serological tests for diagnosis of Lyme borreliosis. Eur J Clin Microbiol Inf Dis.

[48] Oksi J, Uksila J, Marjamaki M, Nikoskelainen J, Viljanen MK (1995). Antibodies against whole sonicated Borrelia burgdorferi spirochetes, 41- kilodalton flagellin, and P39 protein in patients with PCR- or culture- proven late Lyme borreliosis. J Clin Microbiol.

[49] Olsson I, Von Stedingk LV, Hanson HS, Von Stedingk M, Asbrink E, Hovmark A (1991). Comparison of four different serological methods for detection of antibodies to Borrelia burgdorferi in erythema migrans. Acta Derm-Venereol.

[50] Panelius J, Lahdenne P, Saxen H, Heikkila T, Seppala I (2001). Recombinant flagellin A proteins from Borrelia burgdorferi sensu stricto, B. afzelii, and B. garinii in serodiagnosis of Lyme borreliosis. J Clin Microbiol.

[51] Putzker M, Zoller L (1995). Vergleichende Bewertung Kommerzieller Hamagglutinations-, Enzymimmun- Und Immunblottests in Der Serodiagnostik Der Lyane-Borreliose. Klinisches Labor.

[52] Rauer S, Kayser M, Neubert U, Rasiah C, Vogt A (1995). Establishment of enzyme-linked immunosorbent assay using purified recombinant 83-kilodalton antigen of Borrelia burgdorferi sensu stricto and Borrelia afzelii for serodiagnosis of Lyme borreliosis. J Clin Microbiol.

[53] Reiber H, Ressel CB, Spreer A (2013). Diagnosis of neuroborreliosis - Improved knowledge base for qualified antibody analysis and cerebrospinal fluid data pattern related interpretations. Neurol Psych Brain Res.

[54] Rijpkema S, Groen J, Molkenboer M, Herbrink P, Osterhaus A, Schellekens J (1994). Serum antibodies to the flagellum of Borrelia burgdorferi measured with an inhibition enzyme-linked immunosorbent assay are diagnostic for Lyme borreliosis. Serodiagn Immunother Infect Dis.

[55] Ruzic-Sabljic E, Maraspin V, Cimperman J, Lotric-Furian S, Strle F (2002). Evaluation of immunofluorescence test (IFT) and immuno (western) blot (WB) test in patients with erythema migrans. Wien Klin Wochenschr.

[56] Ryffel K, Peter O, Binet L, Dayer E (1998). Interpretation of immunoblots for Lyme borreliosis using a semiquantitative approach. Clin Microbiol Infect.

[57] Schulte-Spechtel U, Lehnert G, Liegl G, Fingerle V, Heimerl C, Johnson B (2004). Significant improvement of the recombinant Borrelia IgG immunoblot for serodiagnosis of early neuroborreliosis. Int J Med Microbiol.

[58] Smismans A, Goossens VJ, Nulens E, Bruggeman CA (2006). Comparison of five different immunoassays for the detection of Borrelia burgdorferi Igm and IgG antibodies. Clin Microbiol Infect.

[59] Tjernberg I, Henningsson AJ, Eliasson I, Forsberg P, Ernerudh J (2011). Diagnostic performance of cerebrospinal fluid chemokine CXCL13 and antibodies to the C6-peptide in Lyme neuroborreliosis. J Infect.

[60] Tjernberg I, Kruger G, Eliasson I (2007). C6 peptide ELISA test in the serodiagnosis of Lyme borreliosis in Sweden. Eur J Clin Microbiol Infect Dis.

[61] van Burgel ND, Brandenburg A, Gerritsen HJ, Kroes ACM, van Dam AP (2011). High sensitivity and specificity of the C6-peptide ELISA on cerebrospinal fluid in Lyme neuroborreliosis patients. Clin Microbiol Infect.

[62] Wilske B, Fingerle V, Herzer P, Hofmann A, Lehnert G, Peters H (1993). Recombinant immunoblot in the serodiagnosis of Lyme borreliosis. Comparison with indirect immunofluorescence and enzyme-linked immunosorbent assay. Med Microbiol Immunol.

[63] Wilske B, Habermann C, Fingerle V, Hillenbrand B, Jauris-Heipke S, Lehnert G (1999). An improved recombinant IgG immunoblot for serodiagnosis of Lyme borreliosis. Med Microbiol Immunol.

[64] Zoller L, Haude M, Sonntag HG (1990). Validity of the standard assays in the serodiagnosis of Lyme borreliosis and effects of methodical modifications. Lab Med.

[65] Albisetti M, Schaer G, Good M, Boltshauser E, Nadal D (1997). Diagnostic value of cerebrospinal fluid examination in children with peripheral facial palsy and suspected Lyme borreliosis. Neurology.

[66] Barrial K, Roure-Sobas C, Carricajo A, Boibieux A, Tigaud S (2011). Evaluation des performances de quatre immunoblots commercialises pour la confirmation de la borreliose de Lyme dans une population de patients suivis en Rhone-Alpes. Immuno-Analyse et Biologie Specialisee.

[67] Bazovska S, Kondas M, Simkovicova M, Kmety E, Traubner P (2001). Significance of specific antibody determination in Lyme borreliosis diagnosis. Bratisl Lek Listy.

[68] Bednarova J (2006). Cerebrospinal-fluid profile in neuroborreliosis and its diagnostic significance. Folia Microbiol.

[69] Bennet R, Lindgren V, Zweygberg Wirgart B (2008). Borrelia antibodies in children evaluated for Lyme neuroborreliosis. Infection.

[70] Blaauw AAM, Van Loon AM, Schellekens JFP, Bijlsma JWJ (1999). Clinical evaluation of guidelines and two-test approach for Lyme borreliosis. Rheumatology.

[71] Blaauw I, Dijkmans B, Bouma P, Van Der Linden S (1993). Rational diagnosis and treatment in unclassified arthritis: How clinical data may guide requests for Lyme serology and antibiotic treatment. Ann Rheum Dis.

[72] Blanc F, Jaulhac B, Fleury M, De Seze J, De Martino SJ, Remy V (2007). Relevance of the antibody index to diagnose Lyme neuroborreliosis among seropositive patients. Neurology.

[73] Cermakova Z, Ryskova O, Honegr K, Cermakova E, Hanovcova I (2005). Diagnosis of Lyme borreliosis using enzyme immunoanalysis. Med Sci Monitor.

[74] Davidson MM, Ling CL, Chisholm SM, Wiseman AD, Joss AWL, Ho-Yen DO (1999). Evidence-based diagnosis of Lyme borreliosis. Eur J Clin Microbiol Infect Dis.

[75] Ekerfelt C, Ernerudh J, Forsberg P, Jonsson AL, Vrethem M, Arlehag L (2004). Lyme borreliosis in Sweden - Diagnostic performance of five commercial Borrelia serology kits using sera from well-defined patient groups. APMIS.

[76] Jansson C, Carlsson SA, Granlund H, Wahlberg P, Nyman D (2005). Analysis of Borrelia burgdorferi IgG antibodies with a combination of IgG ELISA and VlsE C6 peptide ELISA. Clin Microbiol Infect.

[77] Kolmel HW, Neumann P, Lange R (1992). Korrelation Zwischen Neurologischer Erkrankung Und Borrelia-Burgdorferi-Antikorpern in 800 Serum/Liquorpaaren. Nervenarzt.

[78] Ljostad U, Okstad S, Topstad T, Mygland A, Monstad P (2005). Acute peripheral facial palsy in adults. J Neurol.

[79] Popperl AF, Noll F (2000). Diagnostic value of serological test-systems used in diagnosis of infection with Borrelia burgdorferi. [German] Diagnostische Wertigkeit serologischer Verfahren bei der Diagnostik einer Infektion mit Borrelia burgdorferi. Lab Med.

[80] Roux F, Boyer E, Jaulhac B, Dernis E, Closs-Prophette F, Puechal X (2007). Lyme meningoradiculitis: Prospective evaluation of biological diagnosis methods. Eur J Clin Microbiol Infect Dis.

[81] Skarpaas T, Ljostad U, Sobye M, Mygland A (2007). Sensitivity and specificity of a commercial C6 peptide enzyme immuno assay in diagnosis of acute Lyme neuroborreliosis. Eur J Clin Microbiol Infect Dis.

[82] Skogman BH, Croner S, Forsberg P, Ernerudh J, Lahdenne P, Sillanpaa H (2008). Improved laboratory diagnostics of lyme neuroborreliosis in children by detection of antibodies to new antigens in cerebrospinal fluid. Pediatr Infect Dis J.

[83] Lijmer JG, Mol BW, Heisterkamp S (1999). Empirical evidence of design-related bias in studies of diagnostic tests. JAMA.

[84] Leeflang MM, Moons KG, Reitsma JB, Zwinderman AH (2008). Bias in sensitivity and specificity caused by data-driven selection of optimal cutoff values: mechanisms, magnitude, and solutions. Clin Chem.

[85] Van Burgel ND (2013). Host-pathogen interactions in Lyme disease and their application in diagnostics.

[86] Coumou J, Hovius JW, van Dam AP (2014). Borrelia burgdorferi sensu lato serology in the Netherlands: guidelines versus daily practice. Eur J Clin Microbiol Infect Dis.

[87] Müller I, Freitag MH, Poggensee G (2012). Evaluating frequency, diagnostic quality, and cost of Lyme borreliosis testing in Germany: a retrospective model analysis. Clin Dev Immunol.

[88] Dessau RB, Bangsborg JM, Ejlertsen T, Skarphedinsson S, Schønheyder HC (2010). Utilization of serology for the diagnosis of suspected Lyme borreliosis in Denmark: survey of patients seen in general practice. BMC Infect Dis.

[89] Bossuyt PM, Reitsma JB, Bruns DE, Gatsonis CA, Glasziou PP, Irwig LM (2003). Standards for Reporting of Diagnostic Accuracy. Towards complete and accurate reporting of studies of diagnostic accuracy: the STARD initiative. BMJ.

[90] Coumou J, Herkes EA, Brouwer MC, van de Beek D, Tas SW, Casteelen G, van Vugt M, Starink MV, de Vries HJ, de Wever B, Spanjaard L, Hovius JW (2015). Ticking the right boxes: classification of patients suspected of Lyme borreliosis at an academic referral center in the Netherlands. Clin Microbiol Infect.

[91] De Pauw B, Walsh TJ, Donnelly JP (2008). Revised definitions of invasive fungal disease from the European Organization for Research and Treatment of Cancer/Invasive Fungal Infections Cooperative Group and the National Institute of Allergy and Infectious Diseases Mycoses Study Group (EORTC/MSG) Consensus Group. Clin Infect Dis.

[92] Rutjes AW, Reitsma JB, Coomarasamy A, Khan KS, Bossuyt PM (2007). Evaluation of diagnostic tests when there is no gold standard. A review of methods. Health Technol Assess.

[93] Lord SJ, Irwig L, Bossuyt PM (2009). Using the principles of randomized controlled trial design to guide test evaluation. Med Decis Making.

